# Localized Single-Cell Lysis and Manipulation Using Optothermally-Induced Bubbles

**DOI:** 10.3390/mi8040121

**Published:** 2017-04-11

**Authors:** Qihui Fan, Wenqi Hu, Aaron T. Ohta

**Affiliations:** 1 College of Information Science and Technology, Beijing University of Chemical Technology, Beijing 100029, China; fanqihui@hawaii.edu; 2 Department of Electrical Engineering, University of Hawaii at Manoa, Honolulu, HI 96822, USA; wenqi@is.mpg.de

**Keywords:** cell poration, cell lysis, optothermal, cell manipulation

## Abstract

Localized single cells can be lysed precisely and selectively using microbubbles optothermally generated by microsecond laser pulses. The shear stress from the microstreaming surrounding laser-induced microbubbles and direct contact with the surface of expanding bubbles cause the rupture of targeted cell membranes. High-resolution single-cell lysis is demonstrated: cells adjacent to targeted cells are not lysed. It is also shown that only a portion of the cell membrane can be punctured using this method. Both suspension and adherent cell types can be lysed in this system, and cell manipulation can be integrated for cell–cell interaction studies.

## 1. Introduction

Intracellular components provide crucial information for biomedical research. However, the lysis of cells in bulk blends the intracellular components of all the cells, possibly resulting in misleading data due to averaging [1]. In contrast, single-cell analysis can enable the precise study of intracellular processes, like a cell’s response to stimuli, such as soluble factors, cell–cell contact, and mechanical forces [1,2]. Part of many single-cell analysis procedures is the controlled lysis of specific single cells. The single-cell lysis procedure directly affects the downstream analysis, and depends upon parameters such as the cell lysing speed, throughput, selectivity, and compatibility with other processes. 

There are various single-cell lysis techniques, each with its own advantages for downstream analysis. Chemical lysis uses solutions containing surfactants to solubilize lipids and proteins in the cell membrane, creating pores and eventually lysing the whole membrane. Microfluidic devices have enabled the mixing of the surfactant solutions and single cells to enable single-cell chemical lysis [2,3]. However, chemical lysis takes 2–30 s depending on the lysis solution, and the chemical reagents in the solution that need to be removed before subsequent cell analysis [2]. Electrical lysis is another common single-cell lysis technique, in which the cell membrane is broken down by an electric field of sufficient strength. Single-cell lysis can be realized with microelectrodes in a serial process [2,4], with pre-patterned microfluidic chips in a parallel process [5,6,7], or with virtual microelectrodes induced by light [8,9]. However, the working medium in electrical lysis is usually a sucrose solution with a relatively low electrical conductivity to decrease joule heating and bubble formation [6,10]; this is a significantly different environment from cell culture media. Cell lysis by sonication uses ultrasonic waves to generate sufficient pressure to rupture cell membranes [11], while thermal lysis destroys the cell membrane using heat [12]. These two methods have some limitations for single-cell lysis, since it is difficult to focus ultrasonic or thermal energy to a micrometer-scale localized region, and the induced temperatures may damage proteins [2,10,11]. Mechanical lysis directly uses mechanical force to rupture the cell membrane, as in the ‘nanoknives’ used in microfluidic devices [13,14]. Mechanical lysis can minimize the damage to proteins as compared to chemical lysis, thermal heating, and electrical lysis, although the proteins can be captured in the cell debris, increasing the difficulty of extraction [2,10,13].

Laser energy is promising for lysing localized single cells. Laser pulses are focused near the targeted cells in the cell medium to produce a cavitation bubble [2,10]. The rapid expansion or collapse of the induced cavitation bubble induces a hydrodynamic force that can lyse the cell membrane [15,16,17,18,19]. Laser lysis can be simply integrated into microfluidic chips, since it only requires an optical path to the targeted cell regions, and there is no need for complex channels or microelectrodes. Laser lysis can be a rapid, localized, and parallel process for targeted cells. Laser lysis has been demonstrated on single cells using various microfluidic structures, like a polydimethylsiloxane (PDMS) microchannel to confine the shock wave from laser-induced microbubble, or small holding structures to hold single cells under lysis in position [10,19,20]. These microfluidic structures facilitate the focusing of hydrodynamic force on the cells for lysis, but it limits the locations on the device where cell lysis can occur. 

This paper reports on a more precise and flexible single-cell laser-lysis method, which does not need complex microfluidic structures, and can also be combined with on-chip cell manipulation. This single-cell laser lysis system utilizes microsecond laser pulses to generate size-oscillating vapor microbubbles in a biocompatible medium via an optically-absorbent substrate. During the laser on and off cycles, the microbubbles rapidly expand and collapse repeatedly, thus inducing microstreaming and the accompanying hydrodynamic forces around the microbubble. The induced hydrodynamic force can lyse a targeted single cell positioned above the microbubble. The position of the microbubble can be controlled by manipulating the laser focus spot on the substrate, so each single cell can be selectively and precisely lysed with high resolution. This system can also be used for single-cell manipulation by changing the laser pulse width and the position of laser focus [21]. The single-cell manipulation and patterning technique [22] is helpful for studying intercellular [23] and cell-environment [24] interactions. The integration of cell-patterning with a single-cell lysis system is an important feature for single-cell analysis.

## 2. Setup and Mechanism

### 2.1. Setup for Single-Cell Lysis System

**Figure 1:**
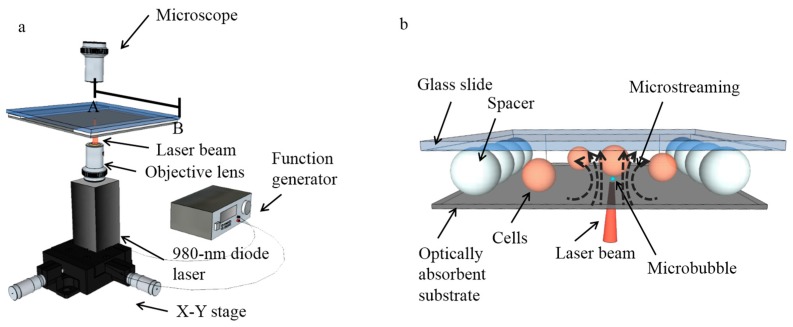
Setup for the single-cell lysis system. (**a**) A 980-nm diode laser was mounted on an *X*-*Y* stage, and modulated by a function generator to produce microsecond laser pulses. The laser light was focused by an objective lens onto the optically-absorbent bottom of the fluidic chamber, creating microbubbles that oscillated in size in the microfluidic chamber. (**b**) 3D structure of the microfluidic chamber filled with biocompatible solutions and consisting of an optically-absorbent substrate, a chamber ceiling made of a glass slide, and polystyrene beads acting as spacers. The cells can be cultured and lysed in the fluidic chamber.

The single-cell lysis system consists of a microsecond-laser-pulse-generation module (Figure 1a) and a fluidic chamber above the laser module, in which cells are lysed (Figure 1b). The microsecond laser pulse was generated by a 980-nm diode laser with a maximum power of 800 mW (Laserlands, 980MD-0.8W-BL, Hengelo, The Netherlands), and then modulated using a function generator (Agilent 33220A, Keysight Technologies, Santa Rosa, CA, USA) to control the on and off cycles via a transistor-transistor logic (TTL) pulse signal. The pulse frequency is set at 50 Hz, while the pulse width can also be controlled precisely for various cell lysis tests. A 10× objective lens was applied to focus the laser onto the bottom of the fluidic chamber from beneath, as a 4.4 µm-diameter light spot, corresponding to a measured intensity of 508 kW/cm^2^. Since the diode laser was mounted on an *X*-*Y* stage, it can be freely moved to locate the laser spot at any position on the bottom of the fluidic chamber bottom for targeting cells.

The fluidic chamber for cell lysis consists of a 1-mm-thick glass slide (top) and an optically-absorbent substrate (bottom). The fluidic chamber was filled with biocompatible solutions as the working media, in which the cells can be cultured and lysed. The optically-absorbent substrate is a 1-mm-thick glass slide, with a 200-nm-thick layer of indium tin oxide (ITO), topped with a 1-μm-thick layer of amorphous silicon (α-silicon). These absorbing materials help the bottom substrate absorb approximately 70% of the incident light from the laser [25], which is converted into heat that induces the vapor microbubbles in the fluidic chamber at the position of the laser spot on the substrate. The top and bottom of the chamber are separated by uniform-sized polystyrene beads (Polysciences, Inc., Warrington, FL, USA) with desired diameters, allowing discrete adjustment of the chamber height. Spacers were put on two opposite sides of the chamber, leaving the other two sides open for the fluid exchange.

### 2.2. Mechanism

**Figure 2:**
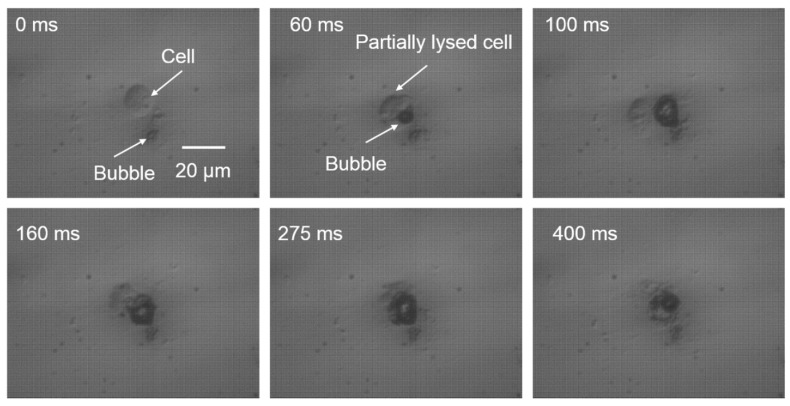
Cell-bubble interaction in one single-cell lysis test. Optical images were taken over a period of 400 ms, corresponding to the length of the cell lysis procedure, at a frame rate of 200 fps.

The light from the focused laser spot on the optically absorbent substrate was transformed into heat, creating a microscale vapor bubble on the bottom of the fluidic chamber. The microbubble rapidly expands when the laser is on, and collapses when the laser is off. This process occurs repeatedly as the laser is pulsed. The size oscillation of the microbubble induced microstreaming around the bubble, corresponding to a strong shear stress. As shown in the Figure 1b, there is a rapid flow in the vertical direction caused by the microbubble oscillation [21,26]. Therefore, the targeted cell above the bubble experiences sufficient shear stress to rupture the cell membrane [17,27]. Another important factor for cell lysis is the direct contact of the cell membrane with the expanding microbubble [28,29]. The expanded bubble can be large enough (diameter of 7 to 14 µm) to contact the cell membrane positioned above the bubble, rupturing the membrane. If the induced microbubble is not large enough to touch the cell membrane, the lysis yield is dramatically decreased. The repeated expanding and collapsing cycles of the microbubble help lyse the whole cell membrane, while one cycle is sufficient to lyse the cell partially. The detailed cell lysis process was recorded with a high-speed camera at a frame rate of 200 fps (Figure 2). The whole cell lysis process lasted 400 ms, during which the membrane of the targeted cell was repeatedly ruptured by the bubble until the cell membrane was completely lysed. 

## 3. Materials and Methods

### 3.1. Cell Culture

NIH/3T3 (murine fibroblasts, ATCC, Manassas, VA, USA) were cultured in Dulbecco’s Modified Eagle’s Medium (DMEM, ATCC), containing 10% bovine serum (Gibco, Invitrogen, Carlsbad, CA, USA), penicillin (100 U/mL), and streptomycin (100 μg/mL). Cells were maintained at 37 °C in a humidified atmosphere of 5% CO_2_ in air. The medium was replaced every 2–3 days. Immediately before cell lysis tests, 1 mL of 0.25% (*w*/*v*) Trypsin-0.53 mM EDTA solution was added to 25 cm^2^ cell culture flask (Falcon, Corning Life Sciences, Corning, NY, USA) to detach the cell monolayer. Then cells were aspirated by gentle pipetting to obtain a cell suspension. The acquired cell suspension was added into microfluidic chamber for the single-cell lysis.

### 3.2. Cell Lysis

Prepared cell suspensions were added into the fluidic chamber, and cells dispersed on the bottom of the fluidic chamber. The laser can be moved freely using the *X*-*Y* stage to target a specific single cell. Once the position of laser and the targeted cell overlapped, the modulated laser pulses were triggered, creating the rapidly expanding cavitation microbubble to lyse the targeted cell.

Calcein AM (Invitrogen) is a green fluorescent dye that can penetrate the membrane of live cell, and emits a green fluorescence when it is hydrolyzed by live cells. If the membrane of a cell containing Calcein AM is ruptured, the cell interior will diffuse into the surrounding medium, and this process can be tracked by monitoring the green fluorescence of the Calcein AM dye. Therefore, prior to the experiment, cells were stained with Calcein AM to indicate cell lysis performance and the distribution of cell cytosol following lysis. 

## 4. Characterization

The lysing efficiency can be affected by various experimental conditions, which were studied to maximize the single-cell lysis rate. The parameters that were varied include: laser pulse width, height of the microfluidic chamber, and the lysis duration for each cell. Thirty cells were tested for each parameter in the following experiments.

### 4.1. Laser Pulse Width

**Figure 3:**
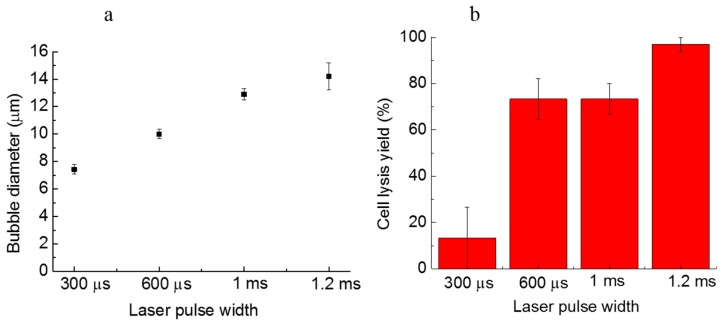
(**a**) The size of microbubbles induced with various laser pulse widths; (**b**) the cell lysis rate as a function of the laser pulse width. More than 30 cells were tested in three parallel experiments. Error bars show the standard error of the measurements.

A longer laser pulse width can induce larger microbubbles, which have higher cell lysis yields. As shown in Figure 3a, the bubble size gradually grows from 7.4 µm ± 0.4 µm to 14.2 µm ± 1.0 µm when the laser pulse width increases from 300 µs to 1.2 ms. With the increasing bubble size, the cell lysis yield is also dramatically increased from 13.3% ± 13.3% to 97.0% ± 3.0% (Figure 3b). It can be seen that, for the 1.2-ms laser pulse width, induced microbubbles with the diameter of approximately 14 µm result in a high cell lysis yield. The tests for various laser pulse widths were all conducted in a 15-µm high microfluidic chamber, using a lysis duration of 400 ms. Thus, when the bubble size is close to the chamber height, there is a higher lysis yield, since the bubble has more chances to contact and rupture the cell membrane. The microstreaming around larger oscillating bubbles also induces strong shear stresses that push the targeted cell against the ceiling of the chamber top, followed by lysis. If the laser pulse is too short, the induced bubble is too small, and may not be able to reach the cell membrane. If the laser pulse width is too long, the size of the induced microbubble will be too large, making it difficult to lyse single cells precisely without damaging nearby cells. 

### 4.2. Microfluidic Chamber Height

The cells can be vertically confined by the microfluidic chamber ceiling to keep them in place while the oscillating microbubble applied shear stress to the cell. Various chamber heights result in different cell lysis performances, depending on how tightly the chamber confines the targeted cells. As the distance between the bubble and the cell membrane increased, the shear stress decreased dramatically. Thus, a controlled chamber height can optimize the working distance between the microbubble and the targeted cell, increasing cell lysis yield.

Empirical observations showed that the minimum chamber height that allowed cell movement in the lateral direction in the fluidic chamber during lysis was 10 µm, which indicates that the cell diameter in the vertical direction is approximately 10 µm. The diameter of NIH3T3 cells was measured to be 16.6 µm ± 1.0 µm (averaged over 10 cells) from the top view when the cells rested on the substrate. However, due to gravitational force, cells in the chamber should have a height less than the diameter, which matches observations. 

**Figure 4:**
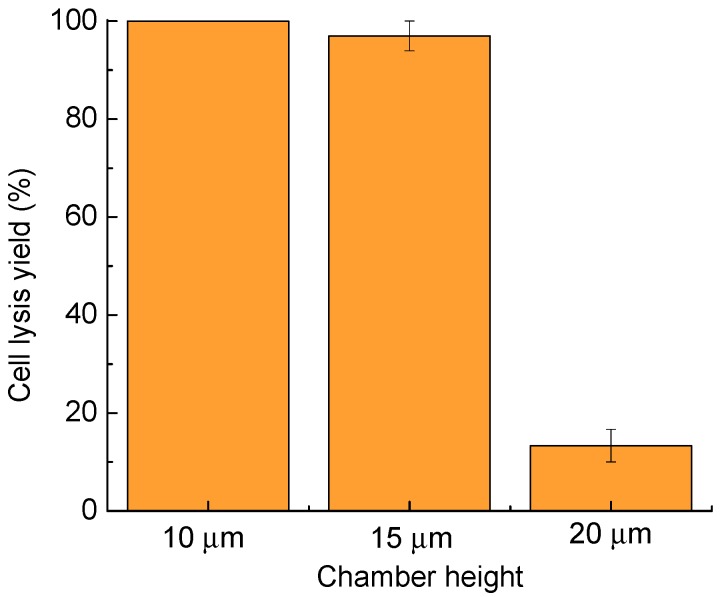
Cell lysis yield as a function of the chamber height. Thirty cells were tested in three parallel experiments for each chamber height. Error bars show the standard error of the measurements.

As described in the setup section, the chamber height can be controlled by the spacer thickness, using polystyrene beads of 10, 15, and 20 µm, respectively (Figure 4). The chamber height of 10 µm provides the highest cell lysis rate, and is similar to the lysis rate of 97.0% ± 3.0% for a 15-µm chamber height. When the chamber height was increased further to 20 µm, the cell lysis yield dropped to less than 20%, due to lack of vertical and lateral confinement of the cells, making it easy for the cells to escape the microstreaming surrounding the bubble. Cells move more freely in the lateral direction in the 15-µm-high fluidic chamber than in the 10-µm-high chamber, which facilitates cell transportation and patterning. Thus, the chamber height of 15 µm was chosen as the optimal fluidic chamber height. In these tests, the laser pulse width was 1.2 ms, and the lysis duration was 400 ms.

Heat produced from the laser absorption in the substrate has previously been characterized [21]. The laser focal point in this work was less than 1 μm in diameter, so even at the maximum laser power, the temperature 14.5 µm away from the center of the laser focal point is less than 32 °C. Since the optimal chamber height for cell lysis was 15 μm, the cells are at a safe temperature, even if the bubble is directly contacting the cell.

### 4.3. Cell Lysis Duration

**Figure 5:**
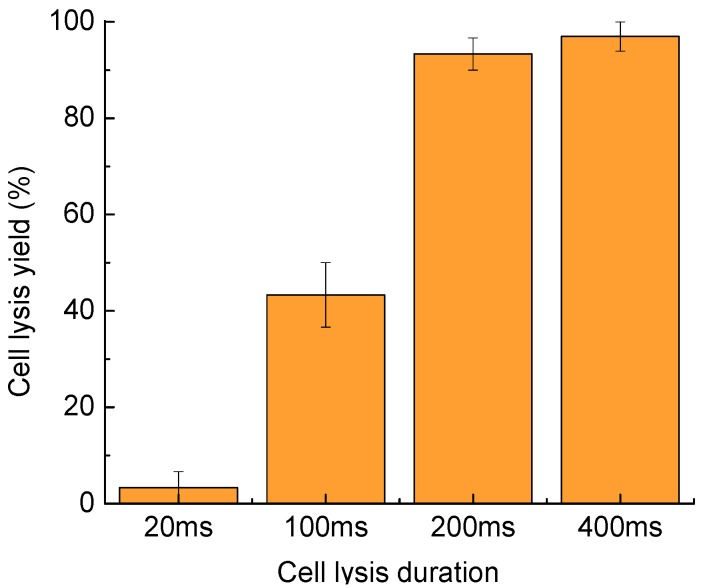
Cell lysis yield as a function of the cell lysis duration. Thirty cells were tested in three parallel experiments. Error bars show the standard error of the measurements.

A longer duration for the cell lysis process enhances the cell lysis yield. However, some studies of highly dynamic processes in cells, like the research on the activity of certain important cell signaling kinases using enzyme-specific peptide reporters [30,31,32], require high temporal resolution, which needs a shorter lysis duration. Therefore, an optimized cell lysis duration minimizes the lysis time per cell while maintaining a high cell lysis yield. As shown in Figure 5, the cell lysis yield can reach to 97.0% ± 3.0% as the cell lysis duration increases from 20 to 400 ms. The targeted cells exposed to laser-pulse-induced microbubbles for 200 ms had thoroughly ruptured membranes at a yield of 93.3% ± 3.3%. As the lysis duration increased to 400 ms, the cell lysis yield also increased, to 97.0% ± 3.0%. Therefore, 200-ms to 400-ms lysis durations result in high cell lysis yields. 

## 5. Results and Discussion

### 5.1. High-Resolution Single-Cell Lysis 

**Figure 6:**
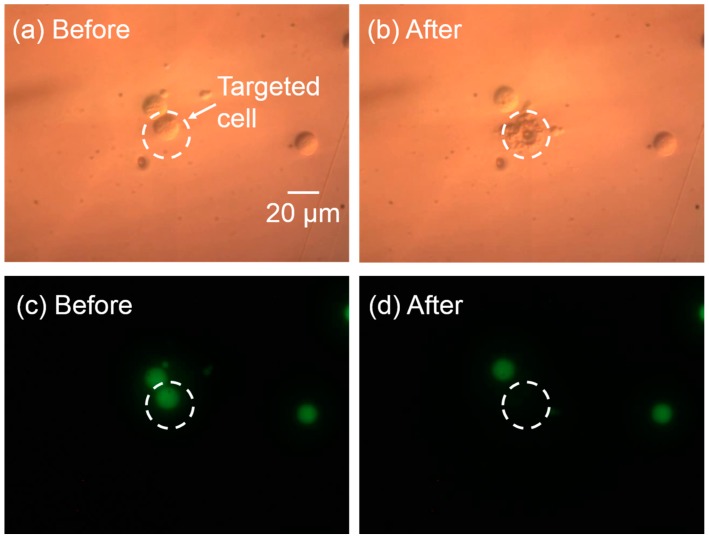
Targeted single-cell lysis result. (**a**,**b**) Differential interference contrast (DIC) images of cells before and after the targeted single-cell lysis. The targeted cell membrane was ruptured, while the nearby cell was kept intact. (**c**,**d**) The corresponding fluorescent images before and after cell lysis. The green fluorescence of the targeted cell dissipated, while the nearby cells maintained their intense green fluorescence, indicating the successful lysis of targeted single cell with no effect on the neighboring cells.

Localized single-cell lysis can be realized precisely using the optimized parameters for this system. To demonstrate high-resolution single-cell lysis, the cell interior was stained with Calcein AM prior to lysis. If the cell membrane was intact, the cell interior was confined, and showed an intense green fluorescence. Once the cell was lysed, the green fluorescence dissipated as the cell interior diffuses out into the solution. As shown in Figure 6, the targeted cell (Figure 6a) was lysed successfully, indicating by the ruptured cell membrane (Figure 6b), and subsequent decrease in the green fluorescence (Figure 6d). The cell adjacent to the targeted cell was not affected. 

### 5.2. Dilution of Cell Content 

**Figure 7:**
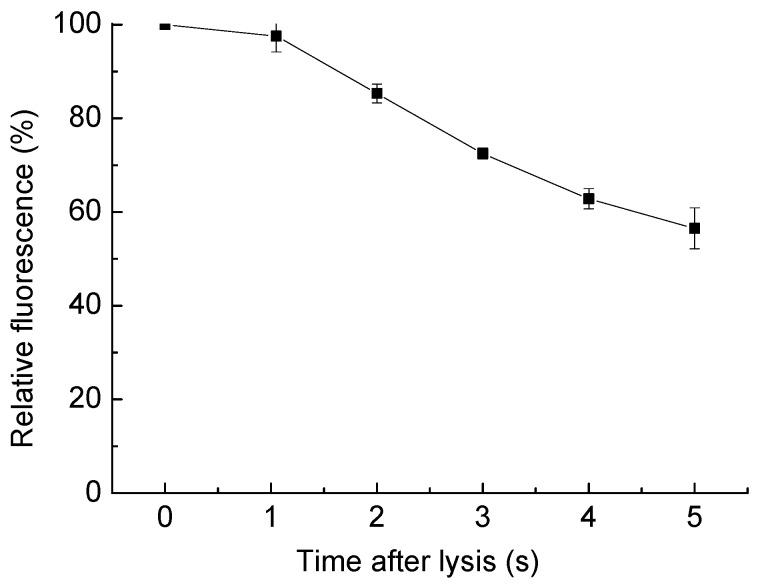
Dilution of cell content as the time passed after cell lysis. Relative fluorescence is measured as the integrated fluorescent density compared with it before the cell lysis, in a 100 µm × 100 µm area surrounding the targeted cell, with the background subtracted. Since the cell interior was stained with Calcein AM, the fluorescence change represents the dilution of cell content. The data is from three parallel tests, and error bars show the standard error of the measurements.

To determine the cell content distribution change after the cell lysis, the cell contents were stained with Calcein AM prior to the tests. The cell was placed in the center of an area encompassing 100 µm × 100 µm. The fluorescent images before cell lysis and 5 s after cell lysis were examined to measure the change of the amount of fluorescent dye, represent the spatial distribution of the cell content. The integrated fluorescence of the examined area was measured with the software ImageJ after the background fluorescence was subtracted. The integrated fluorescence after the cell lysis was compared with the one before cell lysis to quantify the dilution of the cellular contents (Figure 7). After cell lysis, the Calcein AM diffuses, so the integrated fluorescent intensity decreased gradually. However, there was still 85.3% of the fluorescent dye in the examined area surrounding the lysed cell 2 s post-lysis, and 56.5% of the dye left after 5 s, indicating that with this single-cell lysis technique, there is enough time left to collect or analyze the lysate before it disperses widely. Many useful components of the lysate are larger than Calcein AM, like large proteins, genetic materials, or organelles, making these even easier to collect after lysis. 

### 5.3. Lysis of a Subcellular Region

**Figure 8:**
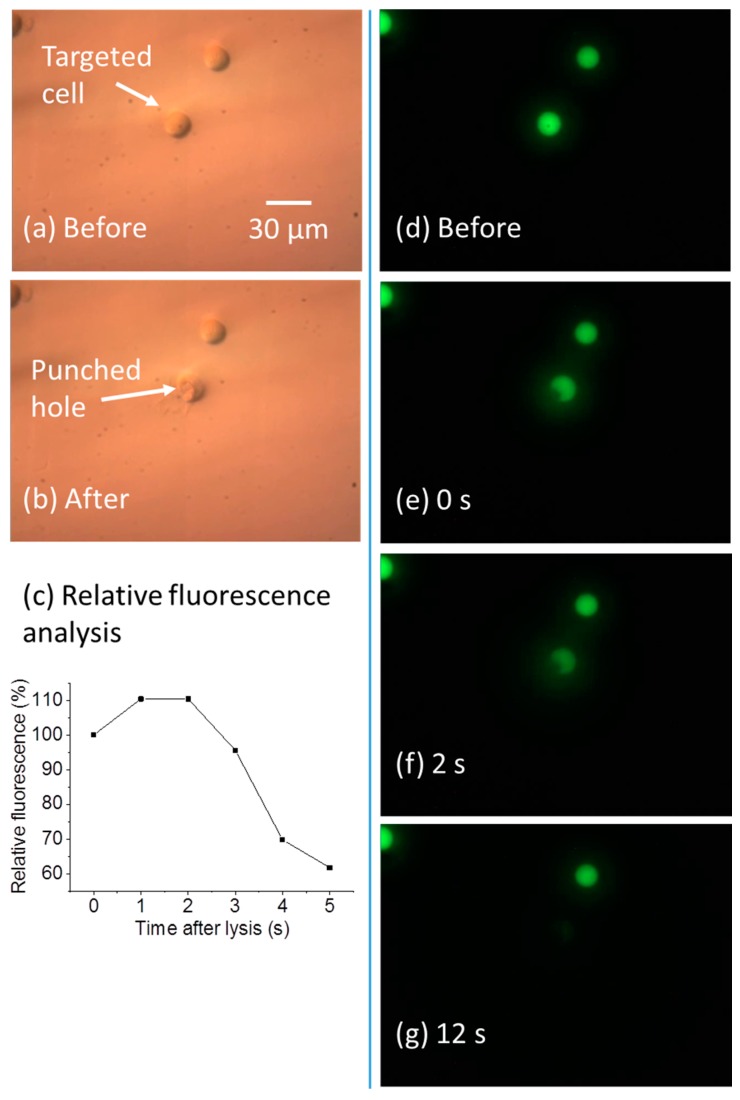
Lysis in a subcellular region. (**a**,**b**) DIC images before and after the subcellular lysis; (**c**) relative fluorescence of the target cell after lysis; (**d**–**g**) fluorescent images before the lysis, and up to 12 s after the lysis.

By controlling the laser pulse width and cell lysis duration, the cell membrane can be ruptured only in a subcellular region. This high-resolution cell lysis can help biological studies, since many biochemical reactions happen in spatially-discrete regions of cells, like the signaling proteins present at the neuronal synapses and growth cone [33]. Thus, lysis in a localized region of the cell can help realize subcellular analysis [33]. As shown in Figure 8a–g, when subcellular lysis occurs, the cell membrane has a localized opening, causing the cell interior to gradually leak out. Figure 8a,b shows the cell morphology change after the subcellular lysis. The membrane was partially ruptured, and a hole was generated. The fluorescent images in Figure 8d–g show the Calcein-AM-stained cell interior gradually leaked out in 12 s after the cell lysis. Figure 8c shows the relative fluorescence of the target lysed cell, measured over a 100 µm by 100 µm area bounding the cell. This result shows the cell contents are confined in the vicinity of the target cell within 5 s, similar to the results for complete lysis. 

**Figure 9:**
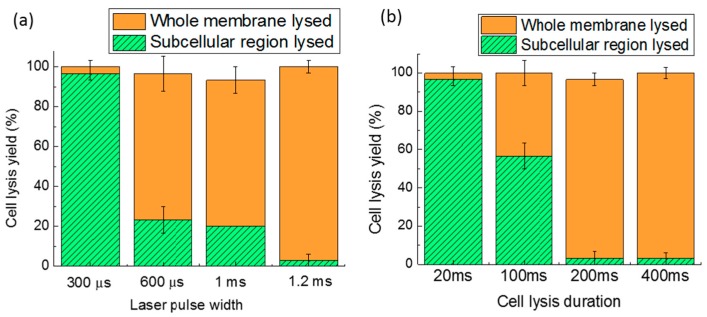
Cell lysis yield as a function of the laser pulse width (**a**); and cell lysis duration (**b**). Thirty cells were tested in three parallel experiments. Error bars show the standard error of the measurements.

Cell lysis in a subcellular region can help with analyzing a specific area of a cell, but it will not release all of the cellular contents completely and rapidly. Though longer laser pulse widths or cell lysis durations can induce lysis over a larger area of the cell membrane, until the whole cell membrane is ruptured, there is always a compromise between lysis resolution and lysis efficiency. Figure 9a,b shows the effect of the laser pulse width and the cell lysis duration on lysis. The bottom portion of each bar represents subcellular lysis, while the upper portion of each bar represents the lysis of the entire cell membrane. The whole bar equals the total cell lysis yield. If the cell was significantly disrupted (more than 50% of the cell shape is disrupted), it was categorized as whole cell lysis, while if more than 50% of the cell shape was maintained, with only a hole appearing in the membrane, it was categorized as subcellular cell lysis. The laser duration was kept at 400 ms to characterize the various pulse width effects, as shown in Figure 9a. For a pulse width of 300 µs, the subcellular lysis approaches 96.7%. When the pulse width was increased to 600 µs, the entire membrane lysis dominates subcellular lysis. Therefore, the pulse width of 300 µs was chosen for subcellular lysis. Another parameter, lysis duration, was varied from 20 to 400 ms (Figure 9b), while keeping the laser pulse width at 1.2 ms. Since the pulse frequency is 50 Hz, only a single laser pulse occurs in the minimum duration of 20 ms, but this is sufficient to induce subcellular lysis. When the lysis duration was lengthened, cells were more likely to be completely lysed. If only the total cell lysis yield is considered, without bias to subcellular or entire cell membrane lysis, the 300-µs laser pulse width and 20-ms lysis duration have the highest cell lysis yield of 100%.

### 5.4. Integration of Cell Manipulation and Cell Lysis

Integration of a cell lysis system to other devices is an important requirement for cell analysis [2,10]. In this single-cell lysis system, it is also possible to perform cell manipulation, cell poration, on-site cell culture, and cell content analysis [21,34]. Cell manipulation prior to cell lysis is useful to studies related to cell–cell interactions, like the influence of stem cells on surrounding fibroblasts [35]. Prior to cell lysis, selected cells can be patterned and co-cultured on-chip for creating cell–cell interactions. A targeted cell in the cell pattern can be lysed precisely, and the lysate can be collected by capillary electrophoresis or analyzed on site via the fluorescent reactions in the medium for the single-cell analysis [18,36]. 

**Figure 10:**
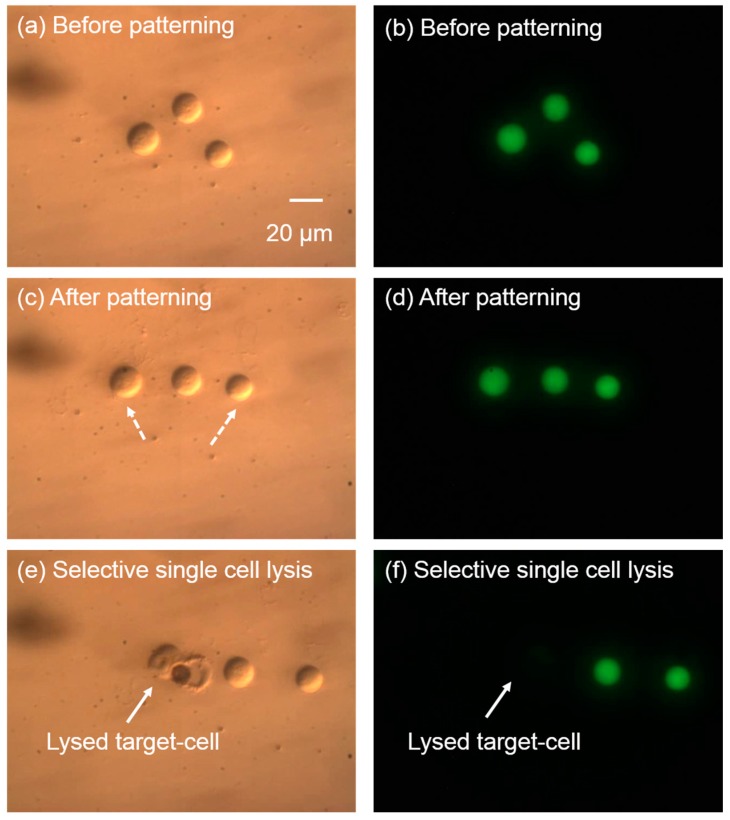
Cell patterning and single cell lysis on the same chip. The left column of images shows the brightfield images, while the right column shows the corresponding fluorescent images. (**a**,**b**) Cells randomly positioned before patterning; (**c**,**d**) cells were patterned into a line with the laser-induced microbubbles. The dashed arrows show the cell trajectories during the cell patterning. (**e**,**f**) The targeted cell was lysed selectively, without affecting neighboring cells.

The integration of the cell patterning and cell lysis in the same microfluidic device has been demonstrated with this single-cell lysis system. As shown in Figure 10, three cells were randomly located. The green fluorescence from Calcein AM in the cell interior indicates intact cell membranes and cell activity. Laser pulse-induced microbubbles were used to manipulate the cells and pattern them into a line via the “pulling mode” of the microbubbles [21] in less than 15 s. This pulling mode creates forces that attracts the cells towards the laser focal point [21], and is achieved by setting the laser pulse width to 7 µs. After cell patterning, the green fluorescence of the cells was maintained, indicating the cell membrane was not affected during the patterning process. When the cell patterning was finished, the left cell was chosen to be lysed. The result in Figure 10e,f shows that the targeted cell was successfully lysed without affecting neighboring cells. This process of cell patterning and cell lysis was fulfilled precisely in the same chip and same place, providing a practical method for biological studies.

### 5.5. Adherent Single-Cell Lysis

**Figure 11:**
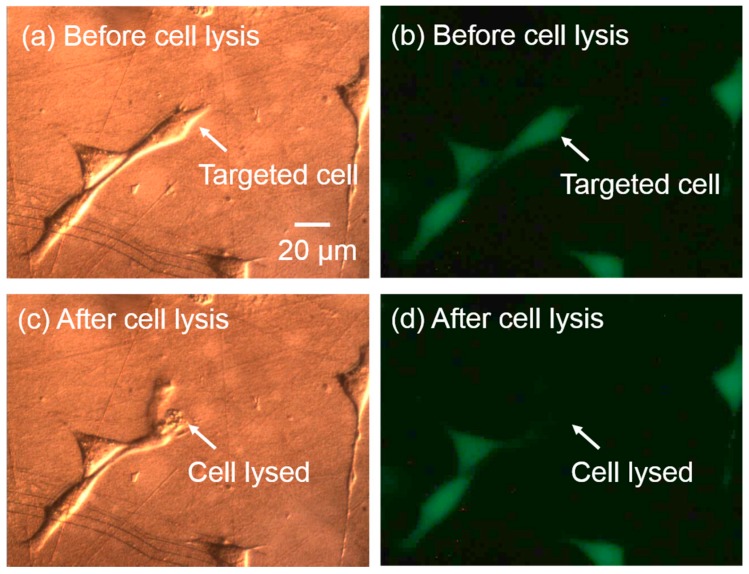
Adherent single-cell lysis. Images in left column are brightfield images, while images in the right column are the corresponding fluorescent images. (**a**,**b**) Before cell lysis, the cell membrane was intact and showing green fluorescence; (**c**,**d**) after cell lysis, the targeted cell was successfully lysed without affecting the neighboring cells.

This single-cell lysis system works both for suspension cells and adherent cells. The cell lysis yield for adherent cells can reach to 100.0% ± 0.0% using the previous optimized parameters, measured in 30 tests. Figure 11 shows a demonstration: after the precise single-cell lysis, the cell membrane was ruptured, and the fluorescent-dye from the cell interior leaked into the medium, while the neighboring cells were not affected. 

## 6. Conclusions

A localized single-cell lysis system using microsecond laser pulses was demonstrated. The microsecond-laser-induced size-oscillating microbubbles apply strong shear stress to the targeted cell right above the bubble. In addition, the rapidly expanding and collapsing bubbles repeatedly directly contact the cell membrane and rupture the membrane. These two factors cause the cell membrane rupture, and complete lysis at a yield of up to 97.0% ± 3.0%. Various laser pulse widths, chamber heights, and cell lysis durations were characterized to obtain higher cell lysis efficiency. Localized single cells can be lysed completely and precisely without affecting the neighboring cells. The lysis can achieve a subcellular spatial resolution. The lysis process takes 300 µs per cell for subcellular membrane lysis and 200 to 400 ms per cell for lysis of the entire cell. After cell lysis, the dilution rate of the of cell content enables subsequent cell lysate collection or on-site analysis, since small molecules in the cell content, such as the fluorescent dye Calcein AM, still retain 85.3% of the original concentration within the surrounding 100 µm × 100 µm area up to 2 s after lysis. Cell manipulation functions can also be integrated into this system, so cells can be freely patterned and co-cultured, and then be selectively lysed for cell analysis. This single-cell lysis system works with suspension cells and adherent cells. 

This single-cell lysis system can be adopted for many biomedical studies, since the experimental setup is easy to acquire and use. There is no need to fabricate complex microfluidic structures on the substrate, and all of the cells in the microfluidic chamber can be selectively lysed with high resolution. Cell lysis and manipulation can occur in a chamber with no microstructures, providing a platform for tissue engineering applications. For example, specific cells can be patterned for tissue growth, followed by the lysis of selected single target cells. This can be used to study cell–cell interaction within the tissue. In the future, the parallel and automated control of microbubbles will be realized, enabling the lysis of multiple target cells at the same time, thus further increasing the cell lysis throughput. This can be realized by a laser scanning system that can project a single laser onto multiple areas of the substrate within one period, as the laser pulse width of 300 μs to 1.2 ms is far smaller than the 20-ms pulse period that was used [25].
